# Multistate Modeling for Determining Transition Probabilities in Sleep Apnea Severity Across Multiple Nights of Recording

**DOI:** 10.1016/j.chpulm.2025.100170

**Published:** 2025-04-08

**Authors:** Jean-Benoit Martinot, Nhat-Nam Le-Dong, Bastien Lechat, Sébastien Bailly, Jean-Louis Pépin

**Affiliations:** a Laboratory of Sleep Medicine, CHU Université catholique de Louvain (UCL) Site Sainte-Elisabeth, Namur, Belgium; b Institute of Experimental and Clinical Research (IREC), Université catholique de Louvain (UCL) Bruxelles Woluwe, Bruxelles, Belgium; c Sunrise, Namur, Belgium; d Flinders Health and Medical Research Institute/Adelaide Institute for Sleep Health, Flinders University, Adelaide, Australia; e University Grenoble Alpes, HP2 Laboratory, Inserm U1300, Grenoble, France; f EFRC Laboratory, Grenoble Alpes University Hospital, Grenoble, France

To the Editor:

OSA is one of the most frequent chronic diseases associated with a high burden on individuals, health systems, and society. In recent years, the internight variability in OSA severity has emerged as a highly intriguing subject in sleep medicine. It was considered primarily as an obstacle to certainty in clinical decision-making because substantial variability is associated with misdiagnosis (20%-60% on any given night) and potentially incorrect treatment indications.^1^ Repeated assessments have been proposed as a solution to mitigate the risk of errors. However, perceptions are shifting toward viewing this variability in severity as a clinical characteristic of OSA, similar to the dynamic progression seen in COPD, asthma, or hypertension. Studies have found that excessive night-to-night variability in OSA severity is also associated with the risk of adverse health outcomes, including higher BP and increased likelihood of atrial fibrillation events.^2^^,^^3^

Advancements in biosignal analytical technology have facilitated the widespread adoption of medical-grade devices for multinight home sleep testing. Among these, mandibular jaw movement (MJM)-based technology has been well recognized for its reliability.^4^

By capturing data across multiple recordings rather than relying on a single snapshot of 1 night’s sleep, longitudinal home sleep testing is expected to provide insightful information about the clinical dynamics of OSA. However, the longitudinal data gathered through such an approach require a more efficient analytical method to determine the transition structure in OSA severity over time.

In a prior investigation,^5^ fluctuations in the MJM-derived apnea-hypopnea index (AHI) were examined by counting transitions across distinct OSA severity levels for individual pairs of nights. However, this approach does not account for variability over longer sequences. We advocate for the adoption of a multistate Markov model (MSM) as a better analytical framework to assess the variability in OSA severity. The MSM approach goes beyond frequency analysis by facilitating the estimation of true transition probabilities across multiple clinical states over a sequence of observations in continuous time. This method is well recognized in medical research for its ability to capture the complex, dynamic evolution of chronic diseases (eg, COPD,^6^ heart failure^7^), but has never been applied in OSA.

The main objective of this study was to determine the transition probabilities among various OSA severity levels in patients with untreated OSA undergoing multiple nights of home-based AHI measurement using MJM analysis (Sunrise).

## Methods

A large data set of 1,225 nights from 475 adults with suspected OSA was acquired between January 2020 and December 2022. All participants provided informed consent for their anonymized data to be used for research (approved by the ethics committee of the Faculty Hospital of Liège University, Belgium: No. 2023/96). Each participant underwent at least 2 nights of recordings (range, 2-9 nights) within a 2-month time window (average interval, 7.8 ± 10.7 days), without any therapeutic intervention. The data acquisition procedure has been previously described.^5^ The main analysis consisted of fitting a multistate model to the input data using the msm package^8^ in the R programming language (R Foundation for Statistical Computing, Vienna, Austria). The input data included patient identifiers, sequential state observations based on 4 levels of OSA severity (1 = non-OSA, 2 = mild OSA, 3 = moderate OSA, 4 = severe OSA), classified by AHI scores at cutoffs of 5, 15, and 30 events/h, and intermittent time points for each state.

## Results

The model demonstrated a satisfactory goodness of fit and allowed for determining the transition probabilities across the 4 levels of OSA severity between any nights. Results from the sensitivity analysis also indicate that the sequential or intermittent observations and the heterogeneity in number of nights among patients do not significantly impact the model’s output.

**Figure 1:**
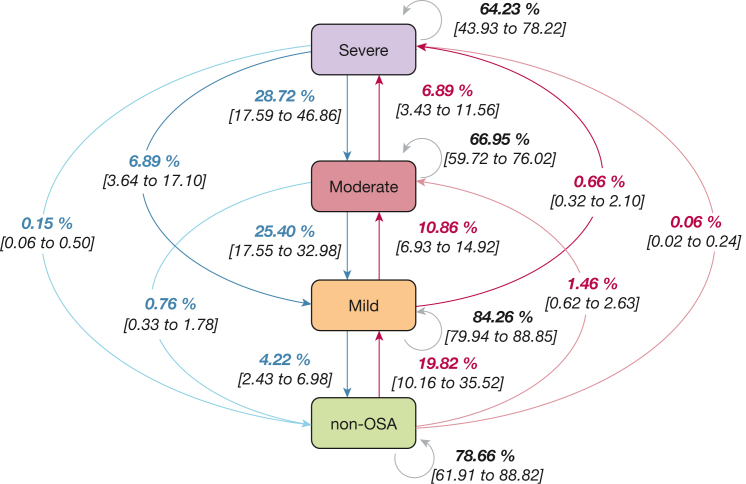
Night-to-night transition probabilities across 4 levels of OSA severity. A network representing the probabilities of transition across 4 levels of OSA severity between any night: non-OSA with AHI of < 5 events/h (green), mild OSA with AHI of 5 to < 15 events/h (orange), moderate OSA with AHI of 15 to < 30 events/h (red), and severe OSA with AHI of ≥ 30 events/h (purple). Transition probabilities and 95% CI were estimated by a Markov multistate model with bootstrapping. The arrows indicate direction of transition: moving to a higher (red), lower (blue), or unchanged (black) OSA severity level. AHI = apnea-hypopnea index.

The characteristics of the study population are detailed in Table 1, and the overall results of transition probabilities are presented in Figure 1. Among participants without OSA on any night, 78.7% maintained an AHI below 5 events/h in subsequent sleep tests, indicating high stability. Transitions from non-OSA to moderate or severe OSA were rare events, as indicated by extremely low likelihood ratios (1.5% and 0.1%, respectively). However, shifting from non-OSA to mild OSA could happen in 19.8% of cases, indicating again the risk of misdiagnosis when decisions are based on a single night’s test. Individuals with severe or moderate OSA show the lowest probabilities of remaining in the same state (64.2% and 67.0%, respectively). The probability of transitioning from moderate to mild OSA was 25.4%, suggesting a substantial level of uncertainty in making therapeutic decisions at the critical AHI threshold of 15 events/h. Transitions from mild to moderate OSA, indicating a worsening clinical condition, exhibited a higher probability than regression from mild to non-OSA levels (10.9% vs 4.2%, respectively). At the most severe OSA states, the probability of transitioning back to mild or normal conditions was low (0.2%-6.9%).

**Table 1:** Characteristics of the Study Population (N = 475)

Parameter	Value
Age, y	48.0 (23.7 to 68.0)
BMI, kg/m^2^	26.5 (19.9 to 39.3)
Neck circumference, cm	39.0 (32.0 to 47.0)
ISI	14.0 (6.0 to 21.0)
ESS	8.0 (2.0 to 19.1)
TST, h	6.7 (4.2 to 8.5)
ArI, events/h	25.6 (13.9 to 48.3)
RDI, events/h	16.2 (5.5 to 46.8)
AHI, events/h	12.2 (4.6 to 41.3)
Non-OSA (AHI < 5)	34 (7.2%)
Mild OSA (AHI 5 to < 15)	266 (56.0%)
Moderate OSA (AHI 15 to < 30)	127 (26.7%)
Severe (AHI ≥ 30)	48 (10.1%)
Sex	
Male	299 (62.9%)
Female	176 (37.1%)
No. of nights	
2	279 (58.7%)
3	143 (30.1%)
4	42 (8.8%)
5 to 9	11 (2.3%)
Average VAHI, events/h	–0.4 (–6.2 to 5.8)
Maximum VAHI, events/h	1.4 (0.0 to 15.4)
Minimum VAHI, events/h	–2.3 (–18.2 to 0.0)

Descriptive results are presented as median (5th-95th percentiles) for numerical data and frequency (proportion) for categorical data. AHI = apnea-hypopnea index; ArI = Arousal Index; ESS = Epworth Sleepiness Scale (0-24); ISI = Insomnia Severity Index (0-28); RDI = respiratory disturbance index; TST = total sleep time; VAHI = night-to-night variation in AHI (estimated for each pair of nights).

Furthermore, the model allows for estimating the time elapsed until the first transition. For example, a patient initially classified as non-OSA may progress to mild OSA in approximately 4 nights (95% CI, 2-8).

## Discussion

Our findings confirm that transitions among OSA severity levels occur with probabilities up to 35%. The results suggest that once a patient’s clinical condition is categorized as severe, spontaneous improvement is unlikely, warranting timely therapeutic intervention. In cases where AHI scores approach critical thresholds, additional sleep tests are advisable to either corroborate the initial findings or detect significant variability, thereby enhancing the accuracy of the classification. Moreover, the variation observed between subsequent tests offers clinically relevant insights (eg, identification of positional endotype variability).

In terms of future perspectives, the MSM approach could offer invaluable insights with significant clinical implications. In epidemiology and health-related cost predictions, this method could enhance the accuracy of forecasting the prevalence of specific clinical states within a population at a given time. Such an approach would be particularly advantageous for conducting real-world, data-driven cost-effectiveness analyses, bypassing the need for simulation processes, as explored in a study on the productivity burden of OSA in the Australian population.^9^

Furthermore, the MSM’s capability to estimate transition probabilities from a normal condition to a more severe form of OSA facilitates the monitoring of acute OSA exacerbations or insufficient control with positive airway pressure treatment in routine clinical settings. Previous studies on positive airway pressure telemonitoring have demonstrated the effectiveness of this approach in clinical practice.^10^

In conclusion, our data demonstrate the feasibility and utility of using a multistate model to explore the dynamic variation in OSA severity categories. Integrating the MSM with continuous at-home AHI monitoring through MJM analysis enables the assessment of variability in OSA severity as a clinical feature. This approach holds significant potential for optimizing clinical practice.

## Funding/Support

J.-L. P. is supported by the French National Research Agency in the framework of the "Investissements d’avenir” program [Grant ANR-15-IDEX-02] and the “e-health and integrated care and trajectories medicine and MIAI artificial intelligence” chairs of excellence from the 10.13039/100019979Grenoble Alpes University Foundation.

## Financial/Nonfinancial Disclosures

The authors have reported to *CHEST Pulmonary* the following: J.-B. M. is a scientific advisor to Sunrise and has been an investigator in pharmaceutical trials for 10.13039/100011096Jazz Pharmaceuticals and Theranexus. N.-N. L.-D. is an employee of Sunrise. B. L. received research grant funding from Withings (paid to institution for work outside the current project), provided in-kind research equipment from Withings (outside of the current project), and is the recipient of an NHMRC Investigator Grant (fund paid to institution for B. L. salary and research support). J.-L. P. is a scientific advisor to Sunrise, has received grants and/or personal fees from ResMed, Philips, Fisher & Paykel, Sefam, AstraZeneca, AGIR à dom, Elevie, VitalAire, Boehringer Ingelheim, Jazz Pharmaceuticals, and Itamar Medical Ltd; and has received research support for clinical studies from Mutualia and 10.13039/100015153Air Liquide Foundation. There was no funding or other financial support for this research from Sunrise. None declared (S. B.).
